# TLR9 Signaling Is Required for the *Porphyromonas gingivalis*-Induced Activation of IL-10-Expressing B Cells

**DOI:** 10.3390/ijms24076693

**Published:** 2023-04-03

**Authors:** Ali Alaqla, Yang Hu, Shengyuan Huang, Sunniva Ruiz, Toshihisa Kawai, Xiaozhe Han

**Affiliations:** 1 Department of Immunology and Infectious Diseases, The Forsyth Institute, 245 First Street, Cambridge, MA 02142, USA; 2 Department of Oral Medicine, Infection and Immunity, Harvard School of Dental Medicine, Boston, MA 02115, USA; 3 Department of Oral Science and Translation Research, College of Dental Medicine, Nova Southeastern University, 3301 College Ave., Fort Lauderdale, FL 33314, USA

**Keywords:** adaptive immune response, TLR-9, periodontal disease, *P. gingivalis*, B10 cells

## Abstract

Immune cell pattern-recognition receptors such as Toll-like receptors (TLRs) play important roles in the regulation of host responses to periodontal pathogens. Our previous studies have demonstrated that immune regulatory B cells were activated by TLRs and alleviated periodontitis inflammation and bone loss. The purpose of this study is to determine the role of TLR9 signaling in the activation and IL-10 production of the primed-immune B cells in vitro. Wild-type (WT) and TLR9 knockout (TLR9KO) mice (C57BL/6 background, *n* = 5) were pre-immunized intraperitoneally with 1 × 10^8^ formalin-fixed *P. gingivalis* and boosted once with 1 × 10^7^ formalin-fixed *P. gingivalis*. Isolated splenocytes and purified B cells from each mouse were cultured with 1 × 10^8^ formalin-fixed *P. gingivalis* for 48 h. Immunocytochemistry was performed to detect CD45^+^ IL-10^+^ cells. Levels of IL-10 expression and secretion in splenocytes and B cells were detected using qRT-PCR and ELISA, respectively. After stimulation with fixed *P. gingivalis*, the percentage of CD45^+^ IL-10^+^ B cells and the level of IL-10 expression were significantly increased (*p* < 0.01) in splenocytes and purified B cells isolated from WT mice. However, these changes were not observed in splenocytes and purified B cells from TLR9KO mice when the cells were treated with fixed *P. gingivalis*. The percentage of CD45^+^ IL-10^+^ B cells was significantly reduced in splenocytes and purified B cells from TLR9KO mice compared to those from WT mice when challenged with *P. gingivalis*. IL-10 expression in B cells from TLR9KO mice was significantly decreased compared to those from WT mice at both the mRNA and protein levels. Additionally, *P. gingivalis*-induced up-regulation of TNF-α mRNA expressions were consistently observed in B cells from both WT and TLR9KO mice. *P. gingivalis*-induced B10 activation and IL-10 production during adaptive responses by primed B cells requires TLR9 signaling and can be achieved independent of T-cell help.

## 1. Introduction

Periodontal disease is an infectious disease initiated by periodontal microorganisms, resulting in the destruction of tooth-supporting tissue and possible tooth loss [1]. The immune-mediated inflammatory response plays a key role in this process. Toll-like receptors (TLRs) are pattern-recognition receptors expressed in immune cells such as macrophages, dendritic cells, T cells and B cells [2]. TLRs play a pivotal role in the mediation of immune responses by recognizing pathogen-associated molecular patterns (PAMPs) that are expressed by different microorganisms. It is now recognized that TLRs could serve as a bridge between the innate and adaptive immune responses [3,4]. The “keystone pathogen hypothesis” proposes that certain oral bacteria can induce host inflammatory responses by interrupting the balance in oral microbiomes and in immune systems [5]. *Porphyromonas gingivalis* (*P. gingivalis*), a potential keystone pathogen, can be recognized by TLRs through various PAMPs and activate innate, adaptive responses leading to periodontal inflammation and bone loss [5,6,7].

B10 cells, a regulatory B cell subset, have recently been extensively studied for their regulatory role in the host immune system during autoimmune and inflammatory conditions [8,9]. Although naturally occurring at a very low frequency (1–3%) B10 cells can be activated and expanded by TLRs and co-stimulatory molecules to regulate innate and adaptive immune responses in infections and autoimmune diseases. The characteristic function of B10 cells is their ability to produce the anti-inflammatory mediator, interleukin 10 (IL-10), which plays a major role in the suppression and resolution of periodontal inflammation [10,11]. In various animal studies, B10 cells have shown robust results in the resolution of inflammation in chronic colitis [12], systemic lupus erythematous [13], collagen-induced arthritis (CIA) [14] and type I diabetes [15,16]. Our recent study using local induction of B10 cells found that the increased gingival expansion of B10 cells and IL-10 production resulted in significant amelioration of periodontal inflammation and bone loss in mice [17]. All these findings strongly suggest that the manipulation of B10 activation and expansion can be a promising therapeutic strategy to control inflammatory diseases.

The interaction between B cells and T cells, together with TLR activation, are essential for the initial development and activation of B10pro cells. However, the role of individual TLR signaling in the adaptive responses involving B10 activation and IL-10 production in primed B cells is currently unclear. The purpose of this study is to determine the role of TLR9 signaling in B10 cell activation and IL-10 production in splenocytes and purified B cells from pre-immunized mice. Our results indicated that *P. gingivalis*-induced B10 activation and IL-10 production by primed B cells requires TLR9 signaling and can be achieved independent of T-cell help.

## 2. Results

### 2.1. CD45^+^IL-10^+^ Cells and IL-10 Expression in Splenocytes and Purified B Cells from WT Mice Were Increased by P. gingivalis Challenge

**Figure 1:**
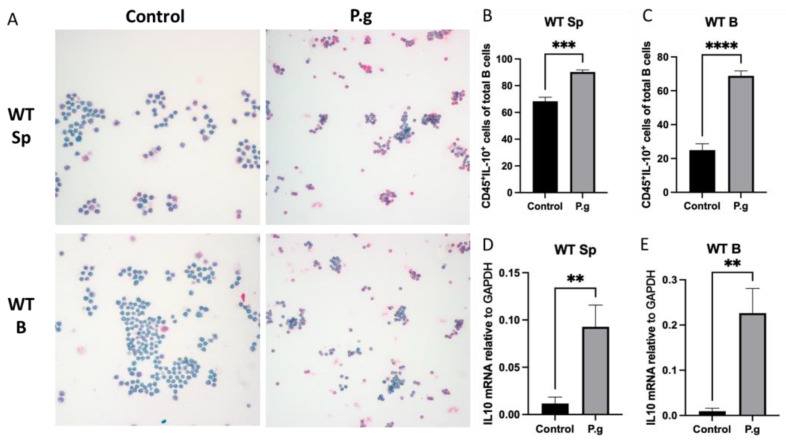
Under *P. gingivalis* stimulation for 24 h, the CD45^+^IL-10^+^ cells and IL-10 mRNA level of splenocytes and B cells isolated from pre-immunized wild-type mice were calculated. (**A**) The representative ICC images of CD45^+^IL-10^+^ cells in each group. CD45+ cells were labeled with rat anti-mouse CD45 antibody conjugated with emerald anti-rat antibody (blue) and IL-10+ cells were labeled with goat anti-mouse IL-10 antibody conjugated with permanent red anti-goat antibody (red). (**B**,**C**) The percentage of CD45^+^IL-10^+^ cells among splenocytes (**B**) and B cells (**C**) were calculated based on cell immunocytochemistry staining. (**D**,**E**) The IL-10 mRNA expression in splenocytes (**D**) and B cells (**E**) were analyzed by quantitative RT-PCR. The data were shown as mean ± SD; significance calculated by unpaired *t*-test was indicated as ** *p* < 0.01, *** *p* < 0.001, **** *p* < 0.0001 (n = 5).

The baseline percentage of CD45^+^IL-10^+^ cells was much lower in purified B cells than splenocytes from *P. gingivalis*-preimmunized WT mice (Figure 1A–C). After being challenged by *P. gingivalis* for 48 h, both cultured splenocytes and B cells from pre-immunized WT mice showed a significant increase in CD45^+^IL-10^+^ cells when compared to controls (Figure 1A–C). The fold increase observed in B cells was much higher than those observed in splenocytes (2.76-fold vs. 1.32-fold). IL-10 mRNA transcript levels were significantly increased in the cultured splenocytes and B cells in the presence of *P. gingivalis* (Figure 1D,E). Such an increase was also found to be greater in treated B cells than those in treated splenocytes (23.84-fold vs. 7.93-fold).

### 2.2. Splenocytes and Purified B Cells from TLR9KO Mice Responded Differently in CD45^+^IL-10^+^ Cells and IL-10 Expression When Challenged by P. gingivalis

**Figure 2:**
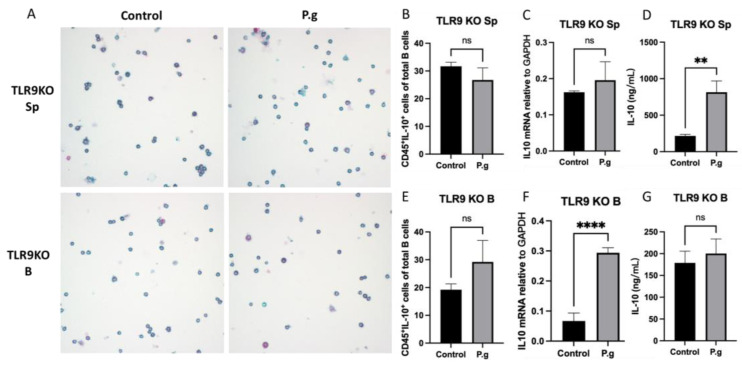
Under *P. gingivalis* stimulation for 24 h, the CD45^+^IL-10^+^ cells, IL-10 mRNA level and secreted IL-10 protein levels of splenocytes and B cells isolated from pre-immunized TLR9 knockout mice were calculated. (**A**) The representative ICC images of CD45^+^IL-10^+^ cells in each group. CD45 cells were labeled with rat anti-mouse CD45 antibody conjugated with emerald anti-rat antibody (blue) and IL-10 were labeled with goat anti-mouse IL-10 antibody conjugated with permanent red anti-goat antibody (red). (**B**,**E**) The percentage of CD45^+^IL-10^+^ cells among splenocytes (**B**) and B cells (**E**) were calculated based on cell immunocytochemistry staining. (**C**,**F**) The IL-10 mRNA expression in splenocytes (**C**) and B cells (**F**) was analyzed by quantitative RT-PCR. (**D**,**G**) The secreted IL-10 protein levels in the supernatants were measured by a mouse IL-10 enzyme-linked ELISA kit using a 1:10 assay diluent in PBST. The data were shown as mean ± SD; significance calculated by unpaired *t*-test was indicated as ** *p* < 0.01, **** *p* < 0.0001, and ns (no significant difference) as *p* > 0.05 (n = 5).

When splenocytes from pre-immunized TLR9KO mice were treated with *P. gingivalis*, no change in the percentage of CD45^+^IL-10^+^ cells was observed compared to control (Figure 2A,B). There were no significant changes in IL-10 mRNA transcripts that can be detected compared to the untreated control group (Figure 2C). However, the IL-10 protein secretion detected by ELISA showed a significant increase in treated splenocytes compared to untreated control (Figure 2D). On the other hand, there were no changes in CD45^+^IL-10^+^ cells percentages when purified B cells were treated with *P. gingivalis* compared to the control group (Figure 2A,E). While IL-10 mRNA transcripts were significantly increased in treated B cells from TLR9KO mice (Figure 2F), no difference in IL-10 protein production was observed between treated and untreated cells (Figure 2G).

### 2.3. Induction of CD45^+^IL-10^+^ Cells and IL-10 Production by Splenocytes and Purified B Cells from TLR9KO Mice Were Compromised upon P. gingivalis Challenge

**Figure 3:**
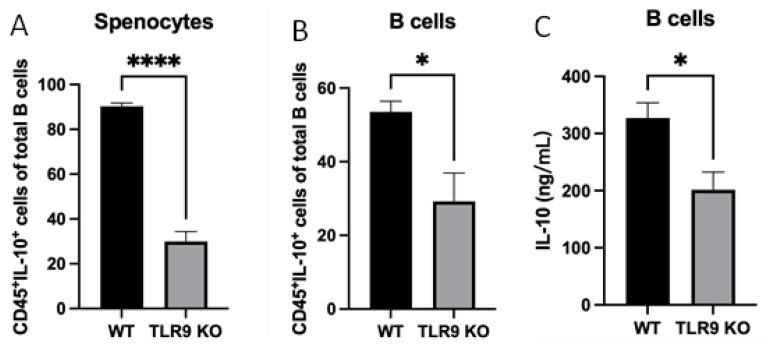
Under *P. gingivalis* stimulation for 24 h, the CD45^+^IL-10^+^ cells of splenocytes and B cells, and secreted IL-10 protein levels of B cells isolated from both pre-immunized wild-type and TLR9 knockout mice were calculated. (**A**,**B**) The percentage of CD45^+^IL-10^+^ cells among splenocytes (**A**) and B cells (**B**) were calculated based on cell immunocytochemistry staining. (**C**) The secreted IL-10 protein levels in the supernatants were measured by a mouse IL-10 enzyme-linked ELISA kit using a 1:10 assay diluent in PBST. The data were shown as mean ± SD; significance calculated by unpaired *t*-test was indicated as * *p* < 0.05, **** *p* < 0.0001 (n = 5).

To determine the role of TLR9 signaling in CD45^+^IL-10^+^ cell formation and IL-10 production, splenocytes and purified B cells were assayed together after treatment with *P. gingivalis* (5 × 10^8^/mL) for 48 h. The percentage of CD45^+^IL-10^+^ cells was significantly lower in cultured splenocytes and B cells from TLR9KO mice compared to those from WT mice after *P. gingivalis* stimulation (Figure 3A,B). Furthermore, ELISA results showed that IL-10 production in cultured B cells from TLR9KO mice in response to *P. gingivalis* stimulation was also reduced compared to treated B cells from WT mice (Figure 3C).

### 2.4. Expression of Inflammatory Cytokine TNF-α Was Not Affected by TLR9 Deficiency

**Figure 4:**
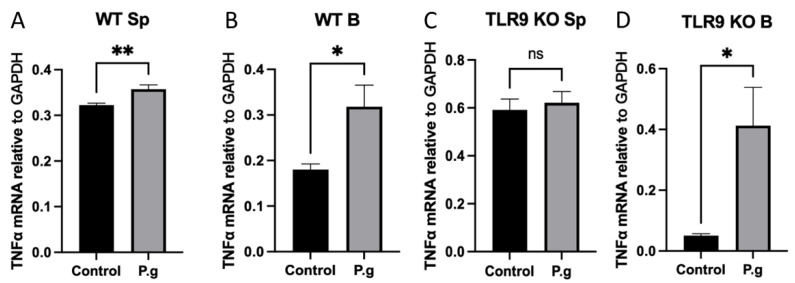
Under *P. gingivalis* stimulation for 24 h, the IL-10 mRNA level of splenocytes and B cells isolated from both wild-type and TLR9 knockout pre-immunized mice were calculated. (**A**) Splenocytes from wild-type mice; (**B**) B cells from wild-type mice; (**C**) Splenocytes from TLR9 knockout mice; (**D**) B cells from TLR9 knockout mice. The data were shown as mean ± SD; significance calculated by unpaired *t*-test was indicated as * *p* < 0.05, ** *p* < 0.01, and ns (no significant difference) as *p* > 0.05 (n = 5).

In order to determine the specificity of the observed B cell response in cultured conditions, the transcript level of TNF-α in cultured splenocytes and B cells was determined by RT-qPCR. The results demonstrated that TNF-α expression was significantly increased in cultured splenocytes and B cells from WT mice when treated with *P. gingivalis* (Figure 4A,B). On the other hand, TNF-α expression did not change in cultured splenocytes from TLR9KO mice but showed a significant increase in cultured B cells from TLR9KO when treated with *P. gingivalis* (Figure 4C,D). These data suggest that TNF-α expression in B cells is TLR9-independent.

## 3. Discussion

TLRs are key elements in the activation of pro- and anti-inflammatory mediators that regulate periodontal inflammation and alveolar bone resorption [18,19,20]. Although the role of TLRs in the recognition of PAMPs and activation of innate and adaptive immune responses through its pro-inflammatory and anti-inflammatory mediators have been extensively studied [21,22], the involvement of TLRs in the adaptive response by a specific regulatory B-cell subset is largely unknown. The exclusive feature that determines B10 cell is its ability to produce anti-inflammatory mediator IL-10 [23]. In our study, we used immunocytochemistry staining of IL-10 together with CD45, a B-cell marker, to estimate potential B10 populations [24]. *P. gingivalis* was selected to prime spleen cells and B cells in mice, as its ability to activate the adaptive immune system has been well documented [25,26]. Among a panel of TLR molecules, TLR9 signaling has been identified as a critical component involved in B10 activation, as well as the expression and secretion of IL-10 [27]. For this reason, we evaluated the role of TLR9 signaling in the activation of B10 cells in the adaptive responses of both WT and TLR9KO mice.

The activation of B10 cells in an inflammatory condition is contributed to by a multifactorial process [11]. Crucial stimulatory molecules that are essential to the proper activation of B10 cells include CD40 and its ligand, CD4^+^ T cells, MHC-class II, LPS, CpG and B cell receptors [9,11]. The latter was deemed a prerequisite molecule in the activation and expansion of B10 cells and acted through an antigen specificity route [28,29,30], yet these findings were exclusive to animals with experimental autoimmune diseases such as systemic lupus erythematosus, experimental autoimmune encephalomyelitis and in injected animal models with chemical solutions such as oxazolone [14,29,31,32]. This antigen specificity route that leads to the activation and expansion of B10 cells through primed WT mice from a microbial pathogen is not clearly understood. Our results regarding the baseline level of B10 cells in untreated splenocytes of WT mice suggested that, in addition to the activation of B10 cells through antigen- specific responses, other immune cell–cell interactions could promote the activation of B10 cells (Figure 1B). In addition, the apparent higher magnitude of B10 expansion and IL-10 expression observed in purified B cells compared to the splenocytes (as reflected by the fold changes) indicated that once primed, B10 activation can be achieved independently of T-cell help (Figure 1B–E).

For the first time, our results showed that primed TLR9KO mice that were stimulated with *P. gingivalis* did not enhance the number of CD45^+^IL-10^+^ cells in spleen cells as well as in B cells (Figure 2B,E), suggesting that B10 expansion is TLR9-dependent. This could be related to the fact that BCR has shown to synergize its effects by colocalizing TLR9 in the endosome [33,34] and the absence of TLR9 is likely to diminish the B10 activation as we observed in the results from TLR9KO mice. Interestingly, the pattern of IL-10 expression at RNA and protein levels in splenocytes was different from what was observed in B cells after treatment. The difference in IL-10 production can only be detected at protein levels in cultured splenocytes (Figure 2C,D), whereas the difference in IL-10 expression could only be observed at mRNA levels in cultured B cells (Figure 2F,G). These results emphasize the notion that discrepancies may exist in transcriptional and translational levels of gene expression during cellular events. There could be two reasons for these observations. First, the level of mRNA for a gene does not always predict its protein levels. For example, Chen et al. reported that protein levels appeared to be strongly correlated with mRNA levels for only a small subset of proteins [35]. The second reason might be related to the targeted fraction of IL-10 measurement between the two analysis approaches that we previously implemented [17]. RT-qPCR was focused on the quantification of total transcripts of IL-10, whereas ICC measures the intracellular IL-10 protein levels but not secreted IL-10. Therefore, the quantitative results from the two experiments may not be directly comparable but rather considered as two separate analyses to assess IL-10 levels.

Since different cells of the innate and adaptive immune system are able to produce potent cytokine such as macrophages, dendritic cells, natural killer cells, T cells and B cells [36,37,38], it is suggested that the deficiency of TLR9 signaling may affect cellular components differently in splenocytes and disrupt IL-10 production in B cells. Nonetheless, our findings clearly indicate that TLR9 plays different roles in the activation of B10 cells in the context of cellular environment.

Reports in the literature indicate that, although a B10 cell is defined by its ability to express IL-10, not every B10 cell is able to express IL-10 highly enough to become an effector B10 cell [30,39]. This is a key concept that we are trying to understand and explore in our research. Using splenocytes along with purified B cells in the experiments allow us to distinguish the B-cell response in the immune microenvironment where cell–cell interaction plays a role, as well as isolated conditions where B-cell-specific responses can be detected. The role of TLR9 in splenocytes could be explained by the fact that other immune cells such as CD4^+^ T cells [40] that interact with B10 cells are critical to the ability of B10 activation and to express higher levels of IL-10 (Figure 3A). Our results also unequivocally demonstrate that B-cell specific response to *P. gingivalis*-induced B10 activation and IL-10 production require TLR9 signaling (Figure 3B,C), and can be achieved independent of T-cell help.

TNF-α is a potent anti-inflammatory mediator and it is very well-studied in the literature as a major cytokine that heavily contributes to inflammatory processes [41,42]. B10 cells are known to express TNF-α, yet in a lower scale compared to IL-10 [43]. It is also reported that IL-10/TNF-α ratio is necessary in order to optimize B10 cell effects on the regulation of the immune system [44]. Hence, we studied the levels of TNF-α expressions in the splenocytes and B cells challenged with *P. gingivalis*. Our results showed that after stimulation with *P. gingivalis* the up-regulation of TNF-α expression was abolished in cultured splenocytes from TLR9KO mice but was consistently up-regulated in cultured B cells irrespective of TLR9 status (Figure 4C,D). These findings suggest that TLR9 signaling mainly impacts TNF-α expression of non-B cells in splenocytes and that TNF-α expression by B cell-specific response is TLR9-independent.

In summary, our study indicated that *P. gingivalis*-induced B10 cell activation and expansion is achieved by antigen-specific adaptive immune responses in a TLR9-dependent manner. Primed B cells alone are able to respond to antigen stimulation for B10 expansion and IL-10 production in the absence of T-cell help.

## 4. Materials and Methods

### 4.1. Bacterial Culture

*P. gingivalis* (ATCC 33277 strain) was grown on anaerobic blood agar plates (NHK agar, Northeast laboratory) in an anaerobic chamber with 85% N_2_, 5% H_2_ and 10% CO_2_. A single colony of *P. gingivalis* was isolated from the plate and grown at ATCC medium 2722. After incubation at 37 °C for 4 days, the number of bacteria in the culture medium was determined by reading optical density values using a spectrometer and comparing them to a curve derived from a standard plate count. Bacteria was collected and fixed with 4% PFA for 30 min at room temperature, then washed three times with phosphate sterile saline (PBS) and re-suspended in PBS at a concentration of 5 × 10^8^/mL.

### 4.2. Animals

Wild-type (WT) and TLR9 knockout (TLR9KO) mice were purchased from Jackson Laboratory (Bar Harbor, Maine, USA) and were used in this study (10 weeks old). All mice used in the study were maintained under specific pathogen-free (SPF) conditions using cages with air circulation systems and maintained at 65–75 degrees Fahrenheit temperature and 50 ± 20% relative humidity. A 14-h light and 10-h dark cycle was maintained. Animals were fed with a standard rodent chow. Mice were pre-immunized with 1 × 10^8^ *P. gingivalis* intraperitoneally on day 0, then followed by 1 × 10^7^ *P. gingivalis* booster injection on day 7. On day 11, the animals were euthanized for spleen and B-cell isolation. The Institutional Animal Care and Use Committee (IACUC) of the Forsyth Institute approved all experimental procedures.

### 4.3. Splenocytes and B Cell Isolation and Culture

Spleen organs were collected in a IMDM + GlutaMAX (Life Technologies, Carlsbad, CA,USA) medium that contained 10% FBS (Atlanta Biologicals, Lawrenceville, GA, USA), 1% penicillin-streptomycin- (Sigma, St. Louis, MO, USA), 2 mM L-glutamine and 2.5 µg/mL amphotericin B (Life Technologies, Carlsbad, CA, USA). Single splenic cells were collected by gentle grinding on a steel mesh and then filtered through a 100 µm cell strainer. Red blood cells were removed by an Ammonium-Chloride-Potassium (ACK) lysis buffer (Life Technologies, Carlsbad, CA, USA) and splenocytes were then resuspended in a complete IMDM medium and filtered with a 40 µm cell strainer. B cells were isolated from splenocytes using a Pan B-cell isolation kit (Miltenyi Biotec, Cambridge, MA, USA). Briefly, single splenic cell suspensions were incubated with biotin-conjugated monoclonal antibodies against non-B cell surface markers (CD4, CD11c, CD49b, CD90, Gr-1 and Ter119) at 4 °C for 10 min followed by incubation with magnetic microbeads conjugated with anti-biotin antibodies at 4 °C for 15 min. Magnetically labeled cells were then depleted by passing through LD columns (Miltenyi Biotec, Cambridge, MA, USA) under the magnetic field of the QuadroMacs separator (Miltenyi Biotec, Cambridge, MA, USA). Unlabeled cells that passed through LD column were collected (contained > 98.5% CD19^+^ cells). Spleen and B cells were counted by hemacytometer and 2 × 10^6^ of cells were cultured in a 200 µL IMDM + GlutaMAX^TM^ complete medium in 24-well plates for 48 h in the presence or absence of 5 × 10^8^/mL fixed *P. gingivalis*.

### 4.4. Immunocytochemistry

Spleen and B cells were fixed with 4% paraformaldehyde for 20 min at RT. Cells were washed twice in PBS and spotted on Superfrost Plus microscope slides (Thermo Fisher Scientific, Hampton, NH, USA). For staining, cells were incubated with 0.25% Triton X-100 for 10 min, blocked with 1% bovine serum albumin (BSA) for 30 min. The cells were incubated with a mixture of rat anti-mouse CD45 Ab (Abcam, Cambridge, UK) diluted at 1:1000 in PBS–Tween 20 (PBST) and goat anti-mouse IL-10 Ab (Santa Cruz) at a dilution of 1:100 for 1 h and washed three times in PBST. A secondary antibody (Ab) double-staining kit (catalog number DS206C; GBI) containing Emerald-conjugated anti-rat Ab and Permanent Red-conjugated anti-goat Ab was used to visualize CD45^+^ IL-10^+^ cells. The stained slides were immediately analyzed using a confocal microscope system (Zeiss LSM780) in a 40× magnification lens. Images of five randomly chosen fields for each cell spot were obtained, and the percentage of CD45^+^ IL-10^+^ cells among all counted cells was calculated (*n* = 5 animals/group).

### 4.5. Quantitative RT-PCR

Total cellular RNA was isolated with pureLink RNA Mini Kit (Life Technologies, Carlsbad, CA, USA) following the manufacturer’s instructions, followed by a purity test using a NanoDrop ND-1000 spectrophotometer (Thermo Scientific, Waltham, MA, USA). RNA was reverse transcribed using SuperScript II Reverse Transcriptase system (Invitrogen, San Diego, CA, USA) and the quantitative real-time PCR was performed in duplicate using the SYBR green I master mix and a LightCycler 480 instrument system (Roche Diagnostics, Indianapolis, IN, USA). The primer sequences were as follows: GAPDH, forward 5′-CCCCAGCAAGGACACTGAGCAA-3′andreverse5′-GTGGGTGCAGCGAACTTTATTGATG-3′;IL-10, forward5′-GACCAGCTGGACAACATACTGCTAA-3′ and reverse 5′-GATAAGGCTTGGCAACCCAAGTAA-3′; TNFα, forward 5′-CACAGAAAGCATGATCCGCGACGT-3′ and reverse 5′-CGGCAGAGAGGAGGTTGACTTTCT-3′. The expressions of the target genes were calculated relative to the GAPDH that acted as an internal control.

### 4.6. Enzyme-Linked Immunosorbent Assay (ELISA)

The secreted IL-10 protein levels in the supernatants of cultured splenocytes and B cells were measured by a mouse IL-10 enzyme-linked immunosorbent assay (ELISA) Max Standard kit (BioLegend, San Diego, CA, USA) using 1:10 assay diluent in PBST. After incubation with 1:500 antibody detection for 1 h, avidin-horseradish peroxidase (Avidin-HRP) and tetramethylbenzidine solutions (TMB) were added for 30 min, and 2N H_2_SO_4_ was used to stop the reaction. For each sample, the assay was performed in duplicate and a standard curve was run with each assay. A micro-plate reader (BioTek) was used to measure the absorbance at 450 nm and 570 nm, and the IL-10 concentration (in picograms per milliliter) was calculated on the basis of the standard curve.

### 4.7. Statistical Analysis

Data analysis was processed using GraphPad Prism (GraphPad Software, version 9.5.0, San Diego, CA, USA). All quantitative data were expressed as means ± SEM. Unpaired Student’s *t*-test was used to analyze differences between two groups. Results with probability values *p* ≤ 0.05 were considered statistically significant.

## Data Availability

The original contributions presented in the study are included in the article. Further inquiries can be directed to the corresponding author.
